# Focal cross transformer: multi-view brain tumor segmentation model based on cross window and focal self-attention

**DOI:** 10.3389/fnins.2023.1192867

**Published:** 2023-05-12

**Authors:** Li Zongren, Wushouer Silamu, Feng Shurui, Yan Guanghui

**Affiliations:** ^1^School of Information Science and Engineering, Xinjiang University, Urumqi, China; ^2^School of Electronic and Information Engineering, Lanzhou Jiaotong University, Lanzhou, China

**Keywords:** brain tumor segmentation, cross window, CNN, Transformer, focal self-attention

## Abstract

**Introduction:**

Recently, the Transformer model and its variants have been a great success in terms of computer vision, and have surpassed the performance of convolutional neural networks (CNN). The key to the success of Transformer vision is the acquisition of short-term and long-term visual dependencies through self-attention mechanisms; this technology can efficiently learn global and remote semantic information interactions. However, there are certain challenges associated with the use of Transformers. The computational cost of the global self-attention mechanism increases quadratically, thus hindering the application of Transformers for high-resolution images.

**Methods:**

In view of this, this paper proposes a multi-view brain tumor segmentation model based on cross windows and focal self-attention which represents a novel mechanism to enlarge the receptive field by parallel cross windows and improve global dependence by using local fine-grained and global coarse-grained interactions. First, the receiving field is increased by parallelizing the self-attention of horizontal and vertical fringes in the cross window, thus achieving strong modeling capability while limiting the computational cost. Second, the focus on self-attention with regards to local fine-grained and global coarse-grained interactions enables the model to capture short-term and long-term visual dependencies in an efficient manner.

**Results:**

Finally, the performance of the model on Brats2021 verification set is as follows: dice Similarity Score of 87.28, 87.35 and 93.28%; Hausdorff Distance (95%) of 4.58 mm, 5.26 mm, 3.78 mm for the enhancing tumor, tumor core and whole tumor, respectively.

**Discussion:**

In summary, the model proposed in this paper has achieved excellent performance while limiting the computational cost.

## 1. Introduction

**Figure 1:**
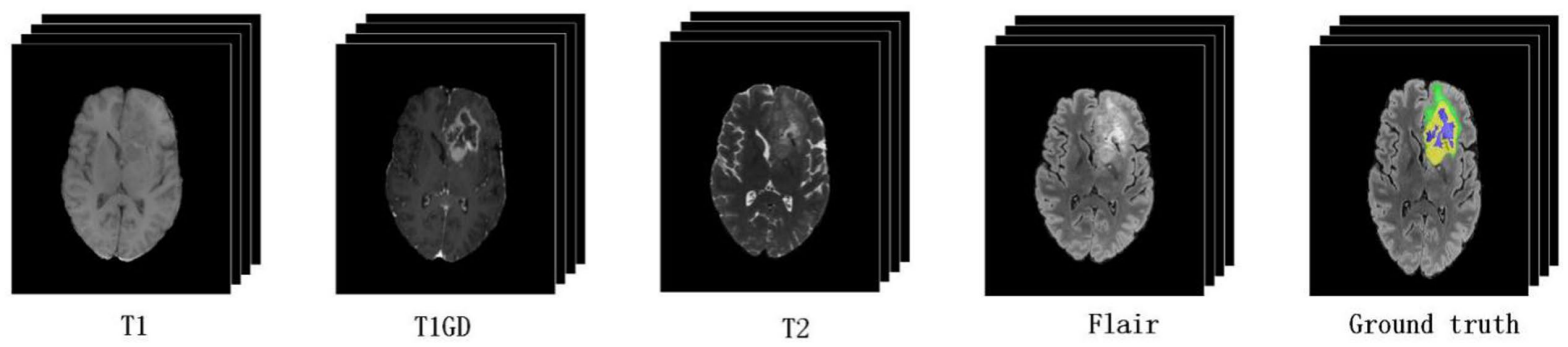
Magnetic resonance imaging (MRI) of multimodal brain tumors. The green, yellow, and blue regions in the ground truth indicate edema (ED), an enhancing tumor (ET), and non-enhancing tumor and necrosis (NCR/NET), respectively.

Brain tumors represent new growths in the cranial cavity that are also known as intracranial tumors and brain cancer and originate from the brain, meninges, nerves, blood vessels and brain appendages, or from other tissues or organs via metastasis. Most of these growths can produce headache, intracranial hypertension, and focal symptoms. The incidence of brain tumors is 7–10 per 100,000 subjects, and more than half of such tumors are malignant. According to a study by the World Health Organization (WHO), brain tumors have become one of the three major tumors endangering human health. The early identification and effective segmentation of brain tumors is very important if clinicians are to formulate treatment plans and improve the survival rates. However, at present, clinicians mainly segment brain tumors from nuclear magnetic resonance imaging (MRI) by hand; this practice is time consuming and also renders the accuracy of segmentation entirely dependent on the experience of the technician or physician. Therefore, convolutional neural networks (CNNs) (Long et al., 2015) and Transformer (Vaswani et al., 2017; Chen et al., 2021; Yuan et al., 2021) and other computer-aided diagnostic technologies are increasingly becoming a new trend with which to segment brain tumor images. Figure 1 shows that MRI data of different morphologies captured different pathological features of tumors.

The segmentation method is based on convolutional neural networks (CNNs) and has generated remarkable achievements in the field of medical image segmentation and other visual fields with its powerful characterization ability. However, CNNs are associated with limitations in global modeling or remote contextual interactions and spatial dependencies prevent further expansion of brain tumor segmentation, thus inspiring the use of Transformer and attention mechanism in medical imaging. Following the pioneering work of Transformer in the field of vision, Vision Transformer (Dosovitskiy et al., 2020) has created a general model in the field of natural language processing (NLP) and vision (Zheng et al., 2021). Several variants were subsequently developed, assisting the introduction of Transformer into medical image classification, target detection, medical image segmentation, and other fields. However, with the prosperity of Transformer in the visual field, many researchers found that although the full attention mechanism of Transformer played a significant role in global modeling or remote context interaction, it also generated problems associated with computational complexity secondary growth (Zhang et al., 2021). Moreover, due to high computational complexity and memory consumption, the full self-attention mechanism of Transformer cannot be applied to medical image segmentation.

To improve efficiency and reduce computational complexity, researchers have suggested replacing the full self-attention mechanism with a limited range of local window self-attention mechanisms. Furthermore, considering the information interaction between windows, shift operation is utilized (Liu et al., 2021, 2022; Cao et al., 2023) and information can be exchanged between nearby Windows, thus alleviating the problem of computational efficiency, at least to some extent. However, expansion of the receptive field in this way is rather slow, and many windows need to be stacked to achieve global self-attention (Liang et al., 2021). For high-resolution image models, such as medical image segmentation, a large receptive field is particularly important as this can affect the local or remote acquisition of contextual information. In view of this, this paper proposes a multi-view brain tumor segmentation model based on cross window and focal self-attention which can retain computational complexity while achieving a large receptive field. Several innovations and major contributions were involved in the development of this new model.

- An innovative mechanism were used to extract characteristic input information from brain tumors, and rich local semantic information was extracted with fine-grained interactions. Then, global semantic information was captured with coarse-grained interactions. This effectively alleviated the problem of high computational complexity associated with the global self-attention mechanism.
- The characteristic information of brain tumor was extracted by cross window, and the self-attention weights within the window were learned from both horizontal and vertical directions by concurrent multiple self-attention mechanisms; then, their weights were concatenated. This expands the receptive field of self-attentional learning and balances the relationship between computational complexity and self-attentional learning ability in Transformer.
- Locally enhanced location coding was adopted to apply the location information to the linear projection value; then, the location information was directly merged into each Transformer block, effectively improving the accuracy of pixel level segmentation for brain tumors.
- The novelty model proposed was applied to the field of brain tumor segmentation and verified on Brats2019 and Brats2021 data sets. The experimental results showed that the model proposed in this paper has achieved excellent performance and outstanding clinical application value.

The sections of this paper are arranged as follows. In the second section, we introduce the existing literature related to this paper. In the third section, we elaborate the architecture of the focal cross window model. The fourth section provides verification of model by using two brain tumor data sets, while the final section summarizes the main contents of this paper and discusses future research and perspectives.

## 2. Related work

### 2.1. Vision Transformer

The Vision Transformer (Dosovitskiy et al., 2020) model, as the first application of Transformer in the field of computer vision, exhibits strong universality, not only in the field of NLP, but also in the field of vision. As far as possible, the model follows the design of the original Transformer model. Firstly, the two-dimensional input feature map was partitioned through the patch partition module, and the partitioned patch was flattened into a token sequence along the channel direction (Chu et al., 2021a,b). A learnable embedded token classification header was added to the original token sequence prior to self-attentional learning; this was implemented by a hidden layer perceptron (MLP) during pre-training (Chu et al., 2021a,b; Touvron et al., 2021; Zhu et al., 2021), implemented by a linear layer when fine-tuned. Because Transformer’s self-attention learning sequence remains constant, it loses important location information. To solve this problem, researchers embedded the location coding information before multi-head self-attention learning. The model uses standard learnable 1D location embedding to preserve the location information in the token sequence. The encoder layer of Transformer is composed of multi-head attention and MLP modules, and the Layernorm (LN) layer is used before each module is applied (Gao et al., 2022; Huang et al., 2022; Lin et al., 2022). The groundbreaking results of the Vision Transformer model demonstrated that a pure Transformer-based architecture can achieve applications comparable to CNNs, thus demonstrating the potential of Vision Transformer for the unified processing of natural language processing and visual tasks. Influenced by the success of the Vision Transformer model, many researchers improved the model from a range of aspects, including computational complexity, segmentation accuracy, and parallelization, so as to improve the efficacy of downstream tasks such as target detection and image segmentation (Howard et al., 2017; He et al., 2021; Wang et al., 2021a,b; Yuan et al., 2021). This led to the development of the Swin Transformer model (Liu et al., 2021) which limits the self-attention learning scope of Vision Transformer to a local window and acquires global information by shifting information between local windows. Thus, the computational complexity of the model is reduced, and the accuracy of image classification is improved. Some researchers combined Vision Transformer with a CNN to connect input features with the Transformer layer after convolution processing, learn local information through CNN, learn global semantic information by Transformer, and then combine the two strategies. This allowed the acquisition of rich semantic feature information. However, when Swin Transformer switches information between local windows during shift operation, the receptive field expands slowly, and many Transformer blocks need to be stacked to obtain global semantic information. However, combining CNN with Transformer (Wang et al., 2021a,b) makes Transformer lose its original ability to capture short-term and remote semantic information at the same time. Therefore, to solve these above problems, we applied the Cross Window to balance the relationship between the computational complexity of the model and the self-attentional learning ability. Under the premise of reducing computational complexity, we expanded the receptive field of self-attentional learning, thus improving the accuracy of brain tumor segmentation.

### 2.2. The global and local self-attention

In the field of medical image analysis, Transformer models usually need to process many long sequence tokens due to the high resolution of images. Over recent years, many researchers have proposed various effective self-attention mechanisms to solve the problem of secondary computing and high memory overhead in Transformer. On the one hand, for many applications featuring medical image segmentation, CNN is combined with Transformer. The token quantity is reduced through CNN down-sampling, and then the global self-attention weight is acquired by coarse-grained interactions. Although this method can improve the efficiency of Transformer, it loses rich semantic information around the tokens, and loses the ability to capture both short-term and remote semantic information. On the other hand, fine-grained self-attention weights are learned in local windows, and then coarse-grained global self-attention weights are captured by window shift or other operations. In this model, we hypothesize that both fine-grained and coarse-grained self-attentional learning are important. Some recently developed advanced models also support his concept (Hu et al., 2018; Bello et al., 2019; Chen et al., 2019; Srinivas et al., 2021). Experimental results of this paper show that the combination of global and local self-attention can effectively improve performance.

**Figure 2:**
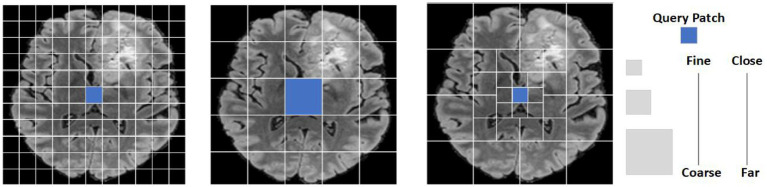
A patch token display of a brain tumor input feature map under different granularity levels. The image on the left shows that feature semantic information is learned by a full self- attention mechanism. The intermediate image representation captures the global semantic feature information completely with coarse granularity. The image on the right shows the proposed model combined fine-grained and coarse-grained focal self-attention mechanisms to capture semantic features.

This paper proposed focal self-attention model is shown in Figure 2. The left image shows that feature semantic information is learned by a full self-attention mechanism which will increase the computational complexity by a factor of two. The middle image indicates that global semantic features are captured by a coarse-grained method. The image on the right shows the proposed model combined fine-grained and coarse-grained focal self-attention mechanism. This mechanism divides patch tokens into three levels of granularity. Self-attentional learning operations of different sizes are performed in each window respectively, thus combining local fine-grained and global coarse-grained strategies to capture short-term and remote semantic information more efficiently and effectively.

## 3. Materials and methods

Focal cross transformer model is a new mechanism to enlarge the receptor field by parallel cross window and improve global dependencies by using local fine-grained and global coarse-grained interactions. The model realizes local and global semantic information interaction by focal self-attention, and uses parallel cross window to enlarge the perceptive field and limit the rapid growth of computational complexity.

### 3.1. Overall architecture

The overall model utilizes UNet encoder decoder architecture, and the encoder architecture of the Focal cross transformer model is shown in Figure 2. Specifically, the input MRI section of multimodal brain tumor data was formulated by


(1) $$X\in {\mathbb{R}}^{H\times W\times D\times C}\;$$

Where the image size is H × W × D, and the number of input channels of the image is represented by C. Firstly, the image was sliced along the depth direction. For each slice, the input size of the image was formulated by


(2) $$X\in {\mathbb{R}}^{H\times W\times 4}\;$$

And then step convolution was used to convert the input image into the patch token of H/4 × W/4. In the encoder path, step convolution was used for down-sampling to acquire the layered architecture. The encoder was divided into four layers; each layer contained N_i_ focal cross transformers. In the focal cross transformer layer, horizontal and vertical stripes were used for parallel self-attention learning, and fine-grained learning was applied around each token. This paper used coarse-grained strategies at long distances to gain global attention. Next, the feature was transformed by feature mapping; in addition, the image size was halved and the number of channels was doubled by step convolution between layers. Then, we stacked the up-sampling and convolution repeatedly to obtain high-resolution segmentation results.

### 3.2. Focal cross transformer

Although the original full self-attention mechanism can capture short-term and remote semantic information, its computational complexity is a quadratic form of feature graph size. To alleviate this problem, many researchers tend to use local windows to limit the scope of self-attentional learning, to reduce the computational complexity and memory consumption. Then, the information between local windows is exchanged by shift operation to acquire global information. However, this operation destroys the ability of the original self-attention mechanism to learn both short-term and remote semantic information at the same time. Furthermore, each token can still only obtain semantic information within a limited area; therefore, more blocks need to be stacked to acquire the global receptive field. The focal self-attention based on cross window would enlarge the receptive field and acquire local and global semantic information interactions in a more efficient manner while limiting the rapid growth of computational complexity.

### 3.3. Focal self-attention

To better realize local and global semantic information interactions, the model used a focal self-attention mechanism that used fine-grained tokens locally and coarse-grained tokens globally, rather than implementing full self-attention mechanism with a fine-grained strategy. Therefore, the global self-attention mechanism can be implemented on the premise of limiting the quadratic increase of computing complexity. Using this system, it was possible to achieve long-term self-attention in less time and with less memory because it only used fine-grained tokens locally and coarse-grained tokens in the long run. However, in practice, we need to query and copy all other tokens for each token, which is still associated with a high computational cost for high-resolution brain tumor images. In view of this, feature mapping was divided into Windows to solve this problem. As shown in Figure 2, the left image represents the use of full self-attention mechanism to learn feature semantic information, which will increase the computational complexity by a factor of two; the middle image represents the use of a coarse-grained strategy to capture global semantic features. However, a large amount of local feature information was lost. The image on the right represents combined fine-grained and coarse-grained focal self-attention mechanism. For the input feature graph Bythe formula(2), this paper first divided data into a window grid of S_P×_S_P_, using fine-grained tokens inside the window and coarse-grained tokens outside the window.

**Figure 3:**
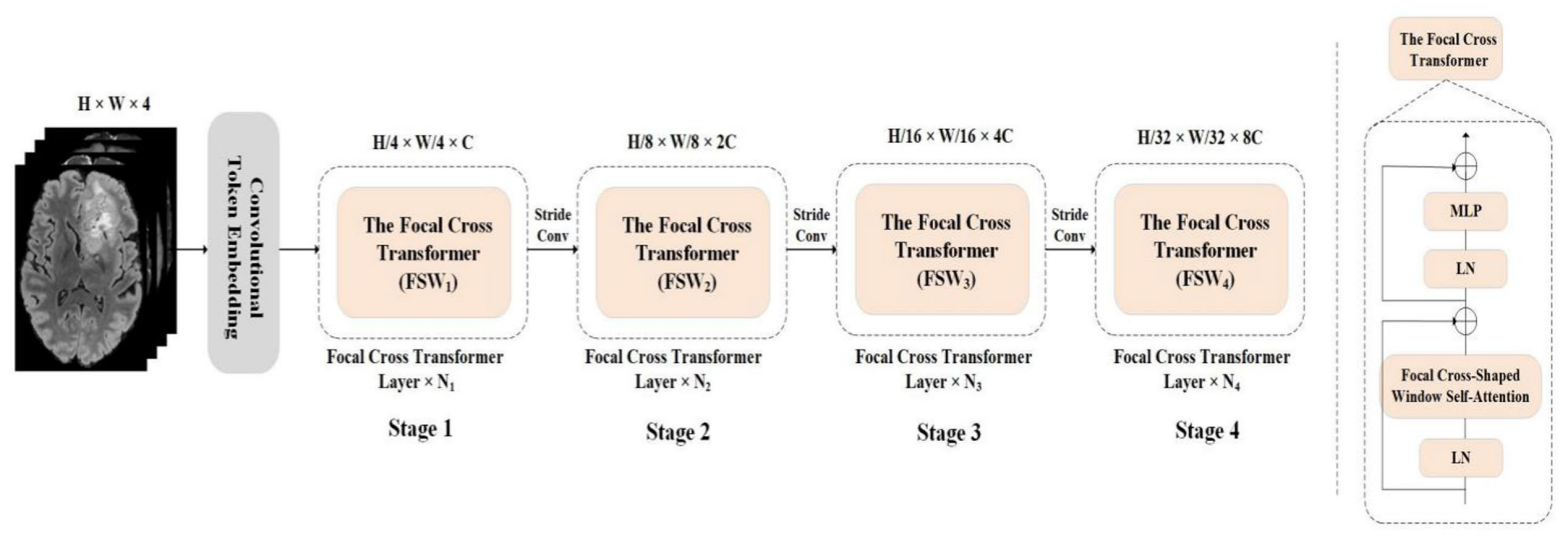
The overall architecture of this paper proposed Focal Cross Transformer. The left image represents the encoder path architecture diagram of the overall architecture, and the right image represents the proposed Focal Cross Transformer.

To express the proposed method more clearly, this paper defined three terms: feature levels, marked with S_L_, which represented the granularity level of extraction for input feature graphs. In Figure 2, this papershow the extraction of three granularity levels. Feature windows size, marked with S_W_, represent the size of the window size in the S_L_ level and the number of summary tokens, thus providing sub-windows. Feature windows number, marked with S_N_, represents the total number of S_W_ in the S_L_ tier. By applying these three terms {S_L_, S_W_, S_N_}, An module that clearly displays the model, as shown in Figure 2 at the fine-grained level; the three tags are identified {3,11,11} Figure 3.

#### 3.3.1. Cross window self-attention

**Figure 4:**
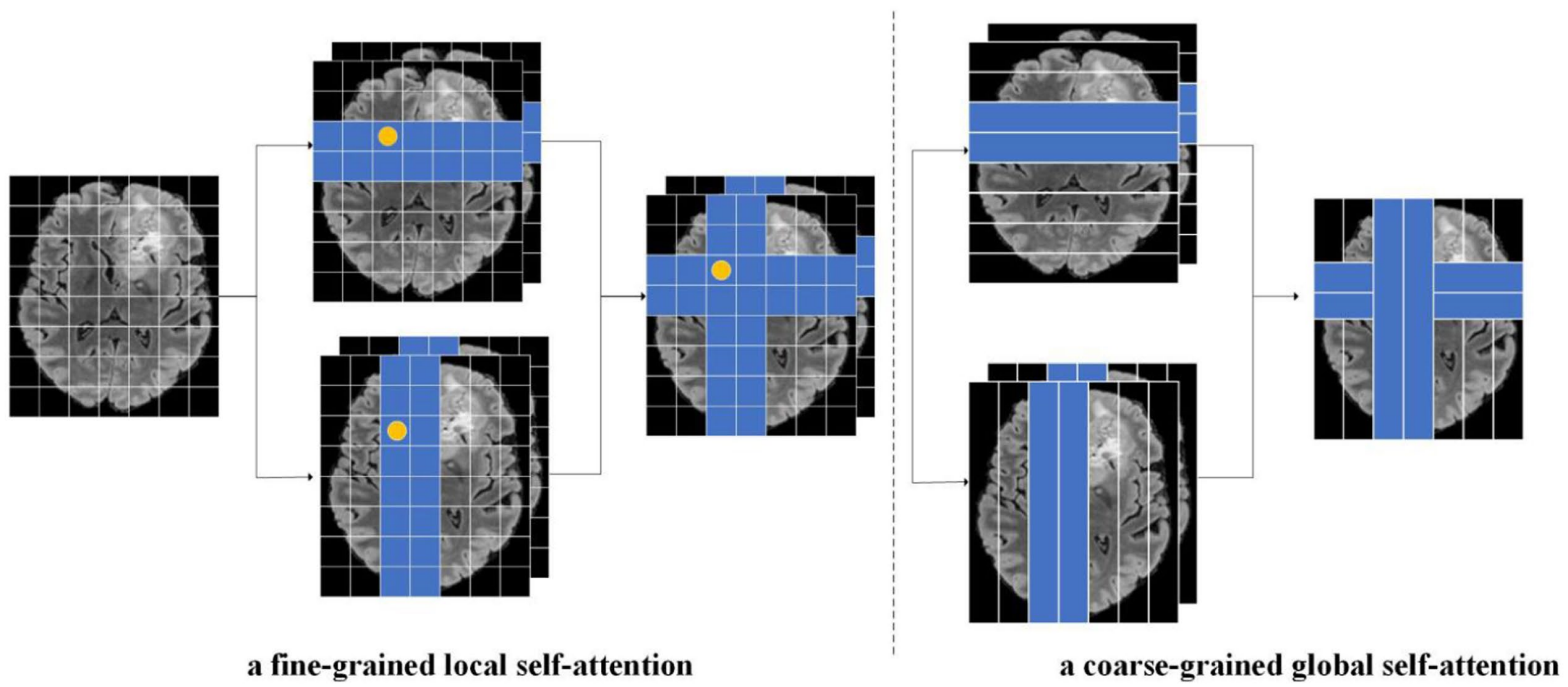
As an illustration of this paper focal cross-attention model, the image on the left represents fine-grained local self-attention while the image on the right represents coarse-grained global self-attention, The input feature X was calculated through fine-grained and coarse-grained strategies. In the fine-grained strategy, the input feature was mapped to the T-head and then the head was divided. Next, we calculated horizontal and vertical autogenous attentions in parallel. Finally, the self-attention results for the two parallel groups in the horizontal direction and vertical directions were cascaded. A similar operation was performed for the coarse-grained strategy.

**Figure 5:**
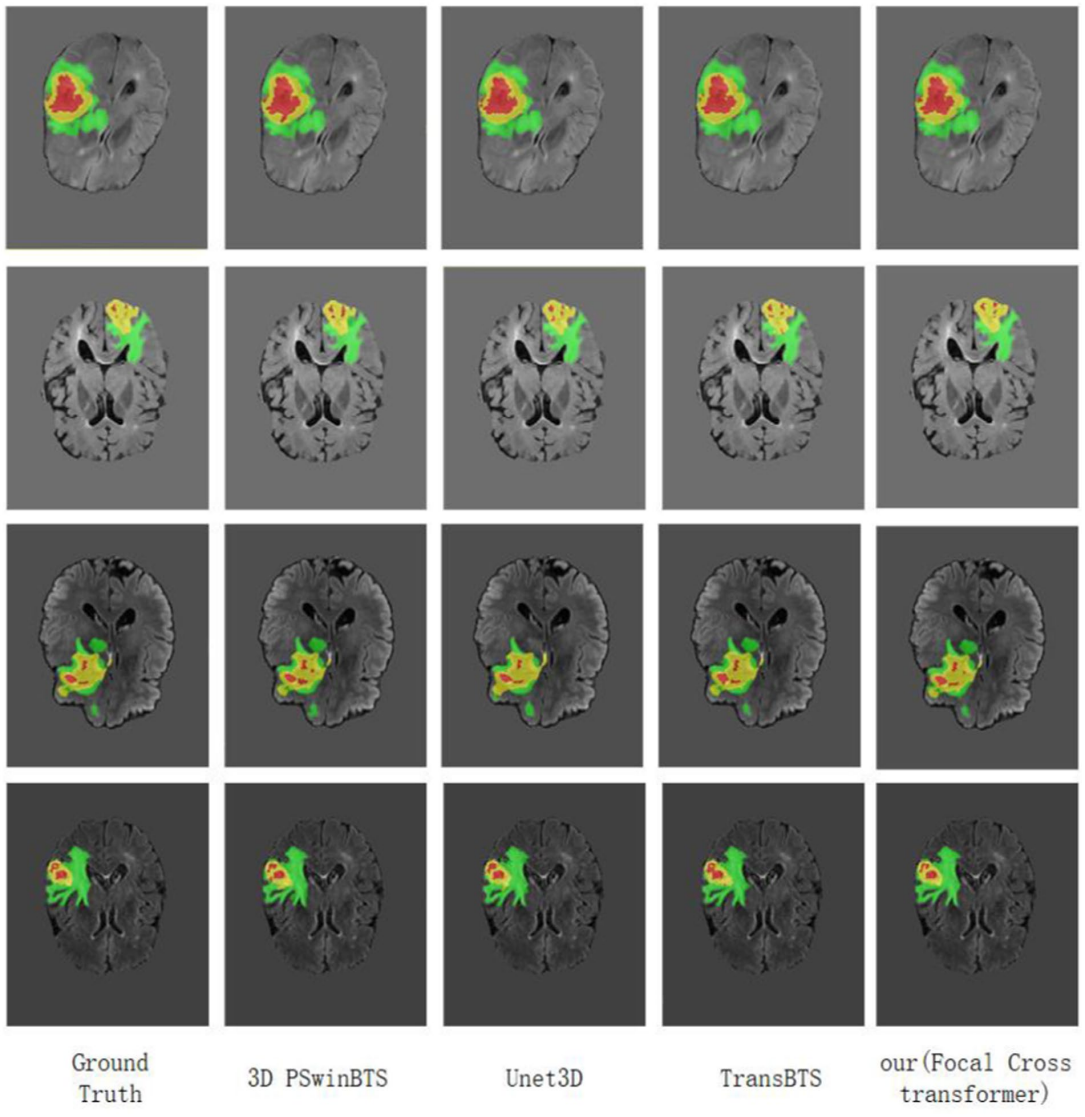
Visualization of MRI brain tumor segmentation under different methods. Focal Cross Transformer was compared with the results derived from Unet3D, 3D PSwinBTS, TransBTS, and other models on the BraTS 2021 dataset.

As shown in Figure 4, this paper separated the features from fine-grained local self-attention and coarse-grained global self-attention. Taking fine-grained local self-attention as an example, a multi-head self-attention mechanism was used to map the input features to T heads; then, each head performed self-attention computations in a horizontal or vertical window Figure 5.

After mapping the input features to T headers, the headers were segmented to realize parallel computation, where {1,2,…,T/2} performs horizontal self-attentional segmentation, {T/2,T/2 + 1,...,T} performs vertical segmentation, and T is usually even. The features were partitioned equally in the horizontal direction and X was partitioned into non-overlapping [X_1_,X_2_,…,X_M_] windows of equal width and S_U_ size. Each window contained S_U_ × W tokens. S_U_ can be used to balance the relationship between self-attention learning and computational complexity, and then fine-grained self-attention weight calculation was carried out for each Token in each S_U_ × W size window. Finally, the self-attention results of two parallel groups in horizontal direction and vertical direction were cascaded.

Let us suppose that the dimensions of queries, keys and values of the input feature X projected to the T-th head are all d_t_; then, the formula for calculating self-attention of the T-th head is as follows:


(3) $$X=\left[X_{1}{\mathrm{,X}}_{2},\ldots {\mathrm{,X}}_{M}\right]\;$$

(4) $$Y_{t}^{i}=\mathrm{Attention}\left(X^{i}W_{Q}^{i}{\mathrm{,X}}^{i}W_{K}^{i}{\mathrm{,X}}^{i}W_{V}^{i}\right)$$

(5) $${\mathrm{Attention}}_{H_{t}}\left(X\right)=\left[Y_{t}^{1}{\mathrm{,Y}}_{t}^{2},\ldots {\mathrm{,Y}}_{t}^{M}\right]$$

(6) $$X^{i}\in {\mathbb{R}}^{\left(S_{U}\times W\right)\times C}$$

(7) $$\;M=\frac{H}{S_{u}},i=\left\{1,2,\ldots \mathrm{,M}\right\}\;$$

(8) $$\;W_{Q}^{i},W_{K}^{i},W_{V}^{i}\in {\mathbb{R}}^{C\times d_{t}}$$

In these formulae, the corresponding vertical window size is similar. The horizontal and vertical parallel grouping results are then cascaded.


(9) $$\mathrm{Focal Cross}-\mathrm{Attention}\left(X\right)=\mathrm{Concat}\left({\mathrm{head}}_{1},\;\;{\mathrm{head}}_{2},\ldots ,\;\;{\mathrm{head}}_{T}\right)$$

### 3.4. Network encoder

Considering that processing the three-dimensional (3D) Transformer will significantly increase computational complexity and memory consumption, we slice the input feature and slice along the depth direction to obtain a two-dimensional image with input feature


(10) $$X\in {\mathbb{R}}^{240\times 240}\;$$

The overlapped convolutional tokens (kernel = 7, stride 4) were then used to obtain the tokens of


(11) $$\frac{H}{4}\times \frac{W}{4}\left(X\in {\mathbb{R}}^{60\times 60}\right)$$

The dimension of each token was C. Then patch token was captured short-term and remote semantic information was acquired through the focal cross transformer layer. In the encoder path, there were four stages, each of which had N_i_ focal cross transformer layers; this maintained the number of tokens. Each focal cross transformer layer was divided into fine-grained and coarse-grained self-attention mechanisms according to granularity level, thus balancing computational complexity and self-attention learning ability according to granularity. At each level of granularity, the self-attention window range was extended by a parallel Cross window; then, the horizontal and vertical self-attention weights were concatenated. To form a hierarchical structure between the focal cross transformer layers, we used a convolutional layer (kernel = 3, stride 2) to reduce the number of tokens and double the channel size. The complete encoder architecture is shown in Figure 3.

### 3.5. Network decoder

To generate segmentation results in the original slice image, we introduced a CNN decoder for up-sampling and to generate pixel-level segmentation. Slice image features


(12) $$X\in {\mathbb{R}}^{\frac{H}{32}\times \frac{H}{32}\times 8C}\;$$

were converted by the feature mapping layer following the encoder layer. Specifically, the sequence data was projected into the standard two-dimensional space through the feature mapping module; then, the image size was expanded and the number of channels was halved by up-sampling through transpose convolution. Then, this paper stacked the upper sampling layer and the convolution layer four times to produce high-resolution segmentation results. Finally, the slices were concatenated to produce segmentation results in the original 3D space.

### 3.6. Positional encoding

Since the sequence order of the self-attention mechanism remained constant, it can lose important positional information. In an ablation experiment performed previously with Swin Transformer (Liu et al., 2021), it was proven that location information can affect the accuracy of image classification; therefore, sresearchers tend to use various location coding mechanisms to re-add the lost location information. At present, absolute position coding, relative position coding and conditional position coding are commonly used. The absolute position code uses sinusoidal functions of different frequencies to generate the code, which is then added to the input. Relative position coding considers the distance between markers in the input sequence and can naturally process long sequences of input information during training. Conditional location coding (CPE) relaxes the limitations imposed by explicit location coding on input size, thus allowing Transformer to handle inputs of different sizes. However, both absolute and relative location coding can add location information to the input token before the Transformer block. This paper concept was derived from the locally enhanced location coding proposed by Dong et al. (2022), in which this model applied location information to the linear projection value and then directly incorporated the location information into each Transformer block.


(13) $$Z_{i}^{t}=\overset{n}{\underset{j=1}{\sum }}\left(a_{\mathrm{ij}}^{t}+b_{\mathrm{ij}}^{t}\right)v_{\mathrm{ij}}^{t}\;$$

In Equation (5), $Z_{i}^{t}$ represents the T th element of vector Z_i_, $a_{ij}^{t}$ represents the result of calculation at the t th element, the queue, key, and $b_{ij}^{t}$ represents position coding information. $v_{ij}^{t}$ represents the value of the self-attention calculation.

## 4. Experimental results

In this paper, Brats2021 and Brats2019 data sets are used to verify the proposed model. Experimental results and ablation experiments demonstrate that the proposed model extends the receptor field by parallel cross window and improves the global dependence by using local fine-grained and global coarse-grained interactions. It can limit the computational complexity and improve the segmentation accuracy of brain tumors.

### 4.1. Training data and pre-processing

#### 4.1.1. Training data

The datasets used for model verification in this study were all Brats datasets. This type of dataset is provided by the brain tumor segmentation challenge organized annually by the Medical Image Computing and Computer Assisted Intervention Society (MICCAI). This challenge has been held for 10 consecutive years and exerts significant influence in the field of medical image segmentation. All imaging data sets are manually segmented by 1 to 4 experienced specialists following the same protocol; then, their markings are reviewed by board-certified neuroradiologists. In the present study, the first dataset we used was Brats2021, which included 2,000 patients (8,000 mpMRI scans) including the training set (1,251 patients), the validation set (219 patients), and the test set (530 patients). Each sample consisted of MRI scans from four modes: native T1-weighted (T1), post-contrast T1-weighted (T1Gd), T2-weighted (T2), T2 Fluid Attenuated Inversion Recovery (T2-flair) volumes, post-contrast T1-weighted (T1GD), T2-weighted (T2), and T2 fluid attenuated inversion recovery (T2-flair) volumes. This paper also included different clinical modalities and a variety of instruments from multiple medical institutions. Each mode had a data size of 240 × 240 × 155 and shared split labels. Each label had four classes {0,1,2,4}: label 0: background; label 1: necrotic tumor core (NCR); label 2: peritumoral edematous/invaded tissue (ED), and label 4: GD-enhancing tumor (ET). The second data set was brats2019, which was not a subset of brats2021; the two datasets were significantly different. The only common data were the images and annotations of BraTS’12-'13; but this did not affect experimental comparisons. The data set included a training set (335 cases) and a validation set (125 cases). The number of samples and modes in each data set were the same.

#### 4.1.2. Pre-processing

All Brats mpMRI scans are available as NIfTI files (.nii.gz).Standardized and enhanced methods were used to process the input data before it was entered into the model for verification. Since the MRI images provided were not standardized, we normalized the gray level of each image and kept the background region as 0. The brats data set has been pretreated with cranial stripping and other procedures. At the same time, four types of data enhancement were implemented in this paper in order to prevent overfitting problems and enhance the Rubon property of the model.

1. Random cropping: considering the large number of black background voxels in the border of the original image, the image was randomly cropped to size (128 × 128 × 128) voxels.
2. Random flip: the image is flipped randomly with a probability of 50% along the coronal plane, sagittal plane and axial plane.
3. Intensity normalization: as the data sets are collected from different instruments in different institutions, the image intensity will be different, and it is necessary to carry out intensity normalization. In this paper, Z-Score normalization is used to process data.

(14) $${\bar{X}}_{j}^{\left(i\right)}=\frac{X_{j}^{\left(i\right)}-\beta_{j}}{\alpha_{j}}\;\;$$

Where β is the mean and α is the standard deviation.

4. Gaussian noise: gaussian noise is added to the training process to improve the robustness and generalization ability of the model. Gaussian noise is a noise generated by adding normal distributed random values with a mean of zero and standard deviation to the input data.

### 4.2. Implementation details and evaluation metrics

#### 4.2.1. Implementation details

This paper trained model with Pytorch, using 8 NVIDIA RTX A5000 (24GB memory) to train 7,050 epochs from scratch using a batch size of 16. For optimization, this paper adopted the Adam optimizer and set its initial learning rate as 0.0003. To achieve more effective convergence, this paper set the decay rate as 0.9 in each iteration. For data set preprocessing, this paper adopted standardization, random flipping, and other strategies to prevent overfitting, but many epochs still needed to be trained. In the training stage, the original training data set was segmented according at a ratio of 8:2 for model training, adjustment, and optimization. According to the inference stage, this paper rescaled the original image and cut the intensity value. Then, this paper uploaded the evaluation model and prediction results to the official website of the host party.

#### 4.2.2. Evaluation metrics

The model used four evaluation metrics for analysis and comparison.

1. The dice similarity coefficient (DSC), which was used to measure the similarity between the brain tumor region predicted by the proposed Focal Cross transformer and the actual segmentation results provided by Brats; the value range was [0,1] and the greater the value, the higher the accuracy of model prediction. Of these, true positive (TP), the actual brain tumor region, was used to predict the brain tumor region; while true negative (TN was predicted to be the normal brain tissue region. The false positive (FP) region was actually normal but was predicted to be brain tumor region. The false negative (FN) region was actually negative but was predicted to be normal.

(15) $$\mathrm{Dice}=\frac{2\mathrm{TP}}{\mathrm{FP}+2\mathrm{TP}+\mathrm{FN}}\;\;$$

2. Hausdorff_95 (95% HD), the Dice coefficient was sensitive to the region inside the tumor, and the Hausdorff distance was sensitive to the delimited boundary. The Hausdorff_95 represents the last value of the Hausdorff distance multiplied by 95% and was used to eliminate the influence of outlier value small subsets.

(16) $${\mathrm{Hausdorff}}_{95}\;\mathrm{distance}=P95\left\{{\mathrm{Sup}}_{x\in Z}d\left(\mathrm{x,Y}\right),\;\;{\mathrm{Sup}}_{y\in Y}d\left(\mathrm{X,y}\right)\right\}$$

3. Sensitivity, it refers to the proportion of pixels whose true value is tumor that are judged as corresponding tumor or edema.

(17) $$\mathrm{Sensitivity}=\frac{\mathrm{TP}}{\mathrm{TP}+\mathrm{FN}}\;$$

4. Specificity, it refers to the proportion of pixels that are judged to be normal among the pixels whose true values are normal.

(18) $$\mathrm{Specificity}=\frac{\mathrm{TN}}{\mathrm{TN}+\mathrm{FP}}\;$$

### 4.3. Main results

#### 4.3.1. Brats 2021

As with previous brain tumor segmentation research, this paper first performed a five-fold cross-validation evaluation on the training set. The average Dice scores of this model for the ET, WT and TC regions were 89.39, 93.58 and 88.65%, respectively. Similarly, at the interface stage, this paper also evaluated the performance of the model by qualitative and quantitative analysis. On the verification set submitted to the official website, we also compared the segmentation results of this model with currently available models; quantitative analysis results are shown in Table 1. The visualized results are shown in Figure 5.

**Table 1:** Comparison and analysis of the BraTS 2021 validation set.

Method	Enhancing tumor (ET)				Tumor core (TC)				Whole tumor (WT)			
	Dice (%)	HD95 (mm)	Sensitivity (%)	Specificity (%)	Dice (%)	HD95 (mm)	Sensitivity (%)	Specificity (%)	Dice (%)	HD95 (mm)	Sensitivity (%)	Specificity (%)
Unet3D (Akbar et al., 2022)	78.02	25.82	80.51	99.97	80.73	21.17	80.55	99.97	89.07	11.78	92.34	99.88
Multi-scale features (Li et al., 2021)	76.89	30.21	78.69	99.97	81.61	16.65	80.57	99.96	90.18	6.16	88.33	99.91
Swin unter (Hatamizadeh et al., 2022)	85.8	6.02	83.68	99.96	88.5	3.77	86.74	99.98	92.6	5.83	93.65	99.95
Evaluating scale attention (Yuan, 2021)	84.79	12.75	-	-	86.55	11.19	-	-	92.65	3.67	-	-
TransBTS	85.16	19.26	83.14	99.97	86.26	12.38	85.74	99.95	91.47	10.62	93.61	99.90
3D PSwinBTS (Liang et al., 2022)	79.48	19.44	81.31	99.95	84.20	7.25	85.11	99.97	90.76	5.57	92.59	99.94
Our (Focal cross transformer)	87.28	4.58	85.62	99.98	87.35	5.26	86.89	99.99	93.28	3.78	94.22	99.97

The Dice scores of this model on the BraTS 2021 validation set for ET, TC and WT were 88.28, 86.35 and 93.28% respectively, and the corresponding results of the Hausdorff were 4.58, 5.26 and 3.78, respectively. Compared with a previous classical algorithm (Table 1), the segmentation accuracy was higher, and the segmentation (in Hausdorff distance) was also significantly improved. Compared with the classical Unet3D model, the Dice coefficient of the model proposed in this paper for the ET, TC and WT areas, was increased by 9.26, 6.62 and 4.21%, respectively. Since the UNet3D model only used a CNN to learn local feature information, its learning ability for global and long-distance semantic features was insufficient, thus resulting in a big difference between the segmentation accuracy and this model. Compared with the TransBTS model combined with Transformer and UNet, the Dice coefficient of the Focal Cross Transformer method for the ET, TC and WT regions, increased by 1.68, 1.09 and 1.81%, respectively. Compared with the Swin Unter model with layered Swin Transformer, the Dice coefficient of the model proposed in this paper for the ET and WT regions increased by 1.48 and 0.68%, respectively, and decreased by 1.15% in the TC region. In the next experiment, we found that adjusting the width of the stripes in focal cross-attention could further improve the segmentation accuracy of the Focal Cross Transformer model in the TC region, but could lead to a large increase in computational complexity and memory. Therefore, this paper adopted the current configuration on the BraTS 2021 dataset for model validation (Table 2).

**Table 2:** Comparison and analysis of the BraTS 2019 validation set.

Method	Enhancing tumor (ET)				Tumor core (TC)				Whole tumor (WT)			
	Dice (%)	HD95 (mm)	Sensitivity (%)	Specificity (%)	Dice (%)	HD95 (mm)	Sensitivity (%)	Specificity (%)	Dice (%)	HD95 (mm)	Sensitivity (%)	Specificity (%)
Unet3D	83.26	23.18	81.32	99.95	82.32	22.28	80.48	99.96	89.58	14.24	91.68	99.88
Swin unter	86.27	7.98	83.39	99.97	89.23	4.68	85.07	99.96	91.23	8.36	92.09	99.91
TransBTS	84.52	17.23	78.69	99.95	85.18	11.27	85.66	99.94	90.21	11.42	92.78	99.89
3D PSwinBTS	81.71	15.63	82.68	99.96	82.46	9.63	84.68	99.97	87.62	7.92	91.85	99.93
Our (Focal cross transformer)	89.68	4.32	85.38	99.97	89.25	3.28	85.96	99.98	93.88	4.26	93.85	99.95

#### 4.3.2. Qualitative analysis

This paper visualized the segmentation results of the model on the BraTS 2021 dataset by applying Unet3D, 3D PSwinBTS, TransBTS and other methods. During visual display, we were unable to obtain the ground truth value for the verification set in the BraTS 2021 dataset; thus, this paper performed five-fold cross-validation evaluation of Unet3D, 3D PSwinBTS, TransBTS, and focal cross Transformer model on the training set.

#### 4.3.3. Brats 2019

this paper also evaluated the segmentation results of model on the BraTS 2019 validation set. Because the BraTS 2019 dataset and the BraTS 2021 dataset are different in terms of the number of cases; the sequence type and image size were the same. This paper directly applied hyperparameters on the BraTS 2021 dataset to train model. The average Dice scores of the Focal Cross Transformer model on the BraTS 2019 validation set for ET, WT and TC were 89.68, 93.88 and 89.25%, respectively. The Hausdorff results were 4.32, 4.26 and 3.28, respectively. Compared with the Unet3D, 3D PSwinBTS, and TransBTS models, the Focal Cross Transformer model showed clear improvements in the Dice coefficient and the Hausdorff two evaluation indices (Table 2).

The model presented in this paper achieves excellent performance on BraTS 2019 validation set. This was mainly because the model uses Fine-grained local self-attention and Coarse-grained global self-attention mechanisms to extract the input characteristic information from brain tumors and extract rich local semantic information through fine-grained grained mechanisms. Then, global semantic information was captured with coarse granularity. This strategy effectively improved the pixel level segmentation accuracy.

### 4.4. Ablation study

To more effectively verify the performance of the model, this paper performed extensive ablation experiments to prove the rationality and feasibility of the model’s design principle. This paper investigated the model’s capabilities in several different ways. Unet3D, 3D PSwinBTS and TransBTS proved that the combination of CNN and Transformer effectively improved the performance of the model. Therefore, this paper no longer independently verified the influence of CNN and Transformer on the performance for brain tumor segmentation.

#### 4.4.1. Coarse-grained global and fine-grained local

This paper used fine-grained tokens locally and coarse-grained tokens globally, rather than implementing a full self-attention fine-grained mechanism. The combination of coarse-grained global self-attention and fine-grained local attention mechanism is an important aspect of the model proposed in this paper. However, full self-attention adopted by vision Transformer cannot be applied to brain tumor segmentation due to high levels of computational complexity. Therefore, it is not possible to verify cases that only use fine-grained full self-attention mechanisms. This paper only verified the comparative performance between a model that adopted the combination of global coarse-grained and local fine-grained mechanisms and a model with the same granularity. This paper use the combined CNN and cross Transformer model in the encoder to perform a comparison experiment between the segmentation of brain tumors with the same particle size and the current model combined with coarse-grained global and fine-grained local mechanisms. The input features size is shown in Formula (1); then, slices were generated along the depth direction. For each slice and the input size of the image is shown in Formula (2), step convolution was used to convert the input image into a patch token of H/4 × W/4. In the encoder path, step convolution was used for down-sampling to achieve the layered architecture. Table 3 shows the results of comparative experiments. For ET, TC and WT, Dice coefficients of the coarse-grained global and fine-grained local models increased by 2.02, 3.03 and 3.69%, respectively.

**Table 3:** Ablation study on coarse-grained global and fine-grained local mechanism.

Method	Dice (%)		
	ET	TC	WT
Coarse-grained	85.26	84.32	89.59
Coarse-grained global and fine-grained local	87.28	87.35	93.28

#### 4.4.2. Cross window

In the model, this paper extended the scope of the self-attention window by applying a parallel cross window and then concatenated the horizontal and vertical self-attention weights. This paper created sw = 1 and sw = 2 Windows separately in the horizontal direction to learn self-attention, and the same configuration was also adopted in the vertical direction; ‘sw’ indicates the size of the sharded self-attention window width. Table 4 shows the Dice coefficients of self-attentional learning and cross window model for ET, TC and WT in the horizontal and vertical directions, respectively. By performing comparative experiments, this paper proved that by combining horizontal and vertical self-attention weights, this model effectively increased the receptive field of the self-attention window and improved the segmentation performance of the model.

**Table 4:** Ablation study on cross window.

Method	Dice (%)		
	ET	TC	WT
Horizontal (sw = 1)	79.38	81.24	83.62
Horizontal (sw = 2)	84.62	85.74	88.49
Vertical (sw = 1)	80.02	82.39	86.27
Vertical (sw = 2)	84.76	86.95	87.83
Cross window	87.28	87.35	93.28

## 5. Conclusion

This paper developed a novel segmentation model for brain tumors. Fine-grained local self-attention and coarse-grained global self-attention mechanisms were combined to extract characteristic input information from brain tumors and extract rich local semantic information through fine-grained mechanisms. Then, global semantic information was captured with coarse granularity. The cross window concurrent multi-head and self-attention mechanism was used to learn the self-attention weight in the window from both horizontal and vertical directions, thus expanding the receptive field of self-attention learning. This also balanced the relationship between computational complexity and self-attention learning ability in Transformer. Experimental results on the Brats2021 and Brats2019 datasets validated proposed model. In future research, we will continue to explore ways to improve Transformer’s global self-attention learning ability and reduce computational complexity so that we can build an efficacious segmentation model for brain tumors.

## Data availability statement

Publicly available datasets were analyzed in this study. This data can be found at: https://www.med.upenn.edu/cbica/brats2021/.

## Author contributions

LZ wrote the main content of the manuscript and carried out experimental research. WS edited and supervised main content of the manuscript. FS and YG put forward suggestions on the structure and experimental part of the paper, and verified by experiments. All the authors reviewed the manuscript and agreed to publish it.

## Funding

This study was supported by Analysis, Prediction and Intervention of Complex Network Behavior in Multilingual Big Data environment, 61433012, National Natural Science Foundation of China, National Key Research and Development Program of Internet Chinese Information Processing and Verification System for Public Security and Social Management, 2014CB340506, automatic segmentation system of brain tumor based on Information Security Technology, 22JR11RA004, Gansu Youth Science and Technology Foundation Program.

## Conflict of interest

The authors declare that the research was conducted in the absence of any commercial or financial relationships that could be construed as a potential conflict of interest.

## Publisher’s note

All claims expressed in this article are solely those of the authors and do not necessarily represent those of their affiliated organizations, or those of the publisher, the editors and the reviewers. Any product that may be evaluated in this article, or claim that may be made by its manufacturer, is not guaranteed or endorsed by the publisher.
